# Looking to a future of improved diabetes management: interview with Professor Steve Bain

**DOI:** 10.4155/fsoa-2016-0069

**Published:** 2016-10-27

**Authors:** Steve Bain

**Affiliations:** 1 Institute of Life Science, Swansea University & Clinical Director, Diabetes Research Unit Cymru, Swansea, Wales, SA2 8PP

**Keywords:** cardiovascular events, diabetes, semaglutide, Type 2 diabetes

## Abstract

**Steve Bain talks to Francesca Lake, Managing Editor:** Steve is currently a Professor at Swansea University Medical School (Wales), Assistant Medical Director for Research & Development for ABM University Health Board and Clinical Lead for the Diabetes Research Unit, Wales. His clinical training included research into the genetics of Type 1 diabetes, with his current clinical interests surrounding exercise in Type 1 diabetes, new therapies and the provision of diabetes services. His background has led him to be Principal Investigator for several multicenter trials, and to be involved in various ethical committees concerning genetics. He led the UK Human Genetics Commission’s report on DNA testing in 2009, and in 2007 was invited to sit on the National DNA Database Ethics Group, established by the Secretary of State for the Home Department. Steve is also a member of the Wales Diabetes & Endocrine Society executive committee and chairs the Specialist Training Committee for Diabetes & Endocrinology for Wales. He also chairs the Board that oversees the Institute of Life Science Joint Clinical Research Facility, the premier clinical research institute in Wales.

## Q Can you tell us about what led you into diabetes research, & how you came to be where you are today?

My wife was one of the first diabetes research nurses in the UK in the mid-1980s and managed to persuade her boss to appoint me as a research fellow in diabetes in Birmingham. She also spotted a newly created Chair in Diabetes in Swansea in the *BMJ* appointments section in 2004 and the rest is history…

## Q What research are you currently working on?

I head up the Diabetes Research Unit Cymru and we have several interests. These include exercise interventions, diabetic eye disease, genetics and new devices for both diabetes monitoring and insulin delivery. I guess my major input is to liaise with large pharmaceutical companies to make sure that our region of Wales gets to be involved in major clinical trials of new therapies.

## Q What implications do you hope this will have for the clinic?

Having experience of new therapies during the trial stages means that we are well placed to be early users when drugs have been licensed and approved for use in Wales. The diabetic retinopathy work is having a real-time impact on how diabetes eye screening is delivered throughout the UK. Meanwhile, the device work leads to new innovations getting to market and will guide future guidelines. Expertise in exercise allows for bespoke advice to be offered in our clinics.

## Q How do you go about ensuring Wales’ input in trials?

We have close working relationships with the major pharmaceutical industries that are involved in diabetes therapies and devices and we maintain good links with the diabetes research networks in the rest of the UK. Then, when we participate in studies we make sure that we recruit quickly, and target and keep high level patient recruitment. This is the best way of ensuring that partners are keen to do further studies in the principality.

## Q Your group is quite proactive about collaborative research, including inclusivity of patient groups. What challenges are there to including patient groups in research, & how do you overcome them?

Creating a patient advisory group has been quite a challenge as you need to get people with the right mix of experience and skills and who also have the time to make a significant contribution. However, the Diabetes Research Unit Cymru has made real strides and is now well-placed to get genuine patient and user input into our diabetes research program.

## Q You have been involved in the continuing debates in DNA ethics. Can you tell us a little about your work with the National DNA Database Ethics Group?

I was a member of the Human Genetics Commission between 2002 and 2009 and during this became the lead for the National DNA Database, which is the UK police database of DNA profiles. This led to me being a co-opted member of the National DNA Database Board and lead on the UK Government report ‘Nothing to hide, nothing to fear? Balancing individual rights and the public interest in the governance and use of the National DNA Database (© Crown copyright 2009). This was very interesting work and a complete change from my usual clinically-based activity.

## Q What are your thoughts on the future of DNA databases & ethical sharing of data?

I think that DNA databases will be an inevitable product of scientific advance and the issue is going to be maintaining confidentiality of data. However, there will always be a balance between personal privacy and public good – for example, most people would accept that a DNA database could be searched if there was the possibility of apprehending a murder suspect. Drawing the line is very difficult.

## Q You were involved in the SUSTAIN 6 trial, for which the results have recently been released. Could you tell us a little about what led to the trial, and its results?

Following on from the controversial withdrawal of a medicine called rosiglitazone from the European market in 2010, all new diabetes medications have to go through large studies to prove that they do not increase the risk of so-called cardiovascular (CV) events (which include heart attacks and strokes). SUSTAIN 6 was one such study [1], which examined the risk associated with an investigational once weekly injectable therapy for diabetes, known as semaglutide. Not only did the trial show that semaglutide was safe, it also showed benefit in terms of reduced numbers of the primary CV outcome of the study. This is only the third modern-day CV study in diabetes to show benefit and was a major surprise given the short duration of the study and the relatively low number of trial participants.

## Q What are the next steps for this research?

Because this was a prelicense study for semaglutide, the regulators in Europe and America may request further studies to confirm this result. Also, there is now a once-daily oral version of semaglutide that is being tested in trials and so that may also need to be scrutinized in a CV study.

## Q What other research should we be looking out for over the next few years in the diabetes arena?

There will be more CV studies that will tell us if all people with Type 2 diabetes benefit from these new diabetes medicines or whether it is only those at highest risk (the group who have been included in the trials so far). We will also see trials combining different drugs that have shown CV benefit to see if they have synergistic effects.

## Q Finally, if you had unlimited funding, what research would you perform, & why?

I would throw money at developing therapies for people with Type 1 diabetes since this is a condition which can dominate people’s lives. There do seem to be novel opportunities to provide patients with islet cell transplants, which could be revolutionary.
